# Engineered butyrate-producing bacteria prevents high fat diet-induced obesity in mice

**DOI:** 10.1186/s12934-020-01350-z

**Published:** 2020-04-25

**Authors:** Liang Bai, Mengxue Gao, Xiaoming Cheng, Guangbo Kang, Xiaocang Cao, He Huang

**Affiliations:** 1 Department of Biochemical Engineering, School of Chemical Engineering and Technology, Key Laboratory of Systems Bioengineering, Ministry of Education, Tianjin University, Tianjin, 300072 China; 2 Department of Gastroenterology and Hepatology, Tianjin Medical University General Hospital, Tianjin Medical University, Tianjin, 300052 China

**Keywords:** Butyric acid, Obesity, High-fat diet, Fecal metabolomics, Engineered bacteria

## Abstract

**Background:**

Obesity is a major problem worldwide and severely affects public safety. As a metabolite of gut microbiota, endogenous butyric acid participates in energy and material metabolism. Considering the serious side effects and weight regain associated with existing weight loss interventions, novel strategies are urgently needed for prevention and treatment of obesity.

**Results:**

In the present study, we engineered *Bacillus subtilis* SCK6 to exhibited enhanced butyric acid production. Compared to the original *Bacillus subtilis* SCK6 strain, the genetically modified BsS-RS06550 strain had higher butyric acid production. The mice were randomly divided into four groups: a normal diet (C) group, a high-fat diet (HFD) group, an HFD + *Bacillus subtilis* SCK6 (HS) group and an HFD + BsS-RS06550 (HE) group. The results showed BsS-RS06550 decreased the body weight, body weight gain, and food intake of HFD mice. BsS-RS06550 had beneficial effects on blood glucose, insulin resistance and hepatic biochemistry. After the 14-week of experiment, fecal samples were collected for nontargeted liquid chromatography-mass spectrometry analysis to identify and quantify significant changes in metabolites. Sixteen potentially significant metabolites were screened, and BsS-RS06550 was shown to potentially regulate disorders in glutathione, methionine, tyrosine, phenylalanine, and purine metabolism and secondary bile acid biosynthesis.

**Conclusions:**

In this study, we successfully engineered *Bacillus subtilis* SCK6 to have enhanced butyric acid production. The results of this work revealed that the genetically modified live bacterium BsS-RS06550 showed potential anti-obesity effects, which may have been related to regulating the levels of metabolites associated with obesity. These results indicate that the use of BsS-RS06550 may be a promising strategy to attenuate obesity.
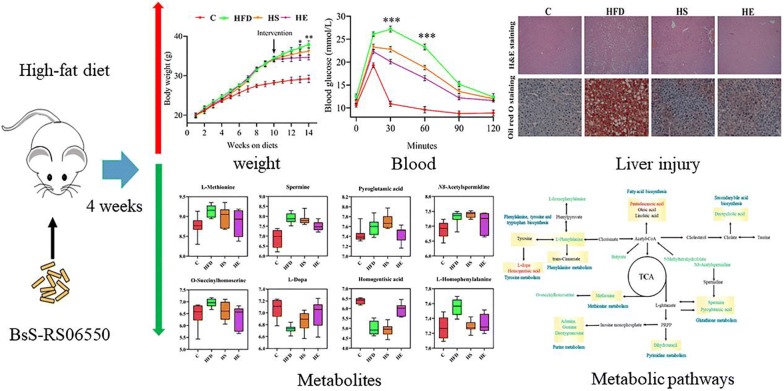

## Background

Obesity is becoming prevalent worldwide and gives rise to a variety of chronic diseases, including metabolic syndrome, type 2 diabetes, and cardiovascular diseases [1, 2]. Thus, obesity is a major public health challenge that places an enormous economic burden on both families and society [3]. Furthermore, the prevalence of obesity in the adolescent and adult reveals the urgent need for effective treatment strategies [4, 5].

Studies have shown that exercise and dietary intervention are effective ways to control and alleviate weight gain [6, 7]. However, it is difficult to maintain long-term exercise or reasonable and healthy eating habits for most patients [8]. Medical therapy and surgery are also possibilities, although these approaches also have serious side effects [9, 10]. Therefore, novel approaches are needed for attenuating obesity.

Metabolic disorders are one of most critical features of obesity, and metabolites and metabolic pathways are closely associated with the occurrence and development of obesity [11–14]. In recent years, researchers have shown that, as metabolites of gut microbiota, short-chain fatty acids (SCFAs) affect the physiological processes of disease by affecting energy supply and immune regulation [15], especially butyric acid (BA), which can alleviate obesity and insulin resistance in mice fed a high-fat diet (HFD) [16]. In addition, BA can reduce appetite and prevent fatty liver via the gut-brain neural circuit [17]. The levels of BA and the microbiota that produce it are significantly reduced in obese individuals compared to healthy subjects, and supplying BA may be a potential strategy for the treatment of obesity [18, 19].

At present, oral butyrate is a commonly used BA supplement but has low bioavailability. In addition, most BA-producing gut microbiota are anaerobic bacteria, which may limit the production capacity of BA, especially in obsess individuals [20]. Probiotics are commonly used as carriers for medicine [21, 22]. *Bacillus subtilis* (*B. subtilis*) is a food-grade probiotic with a clear genetic background and has also been used as an animal feed additive to improve growth performance and immunity [23]. Genetically modified probiotics are increasingly being used as therapeutics for the treatment of obesity [24, 25]. In the present study, *B. subtilis* SCK6 (SCK6) was genetically modified to enhance its production of BA. The purpose of this study was to investigate the potential preventative of the genetically modified *Bacillus subtilis* SCK6 strain (BsS-RS06550) in mice fed an HFD.

## Results

### Genetic modification of *Bacillus subtilis* SCK6 to enhance BA production

**Fig. 1:**
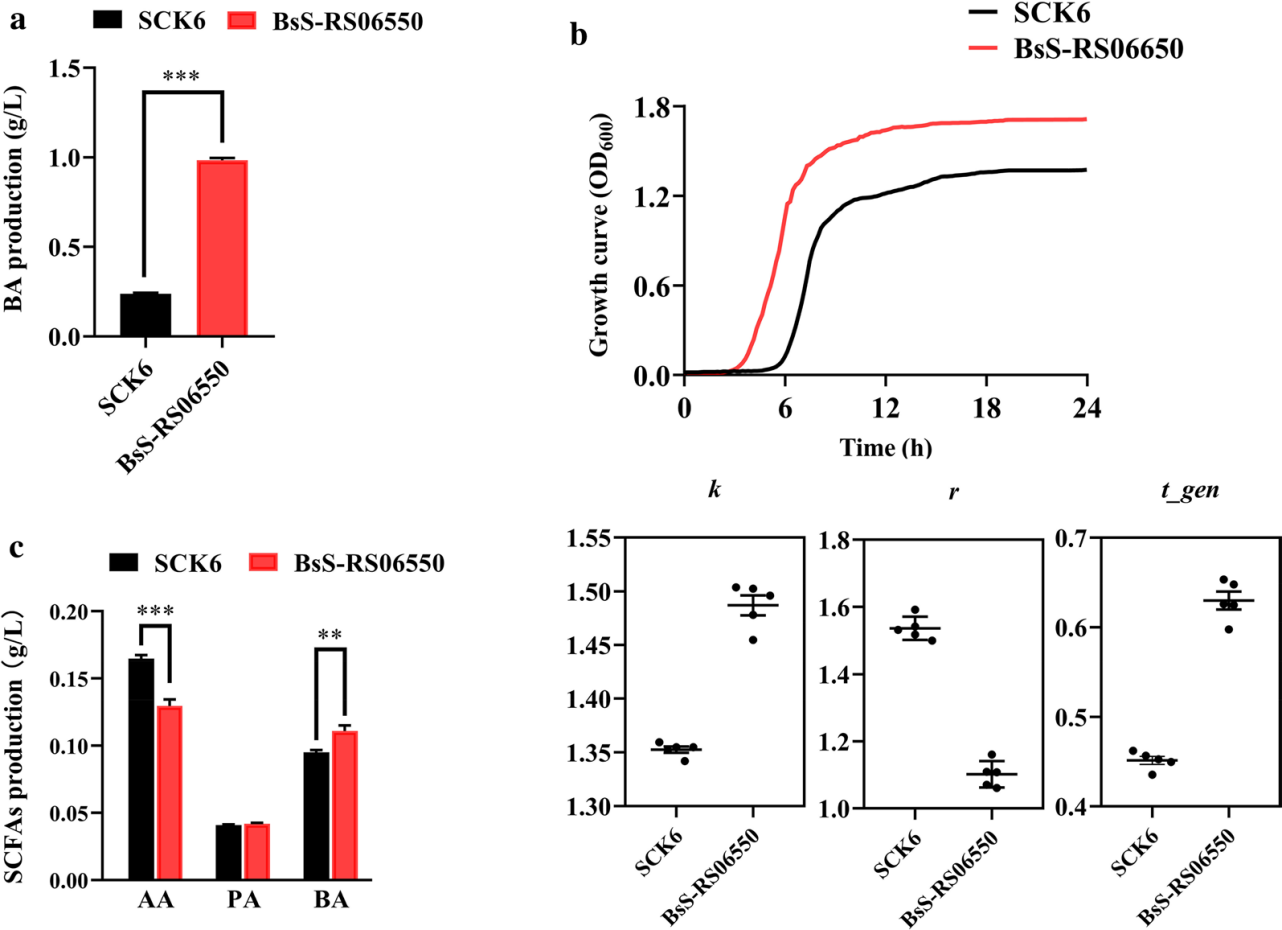
In vitro culture of *B. subtilis* SCK6 and BsS-RS06550. **a** BA production of SCK6 and BsS-RS06550. **b** Growth curves of *B. subtilis* SCK6 and BsS-RS06550 at OD_600_. Growth curve parameters, *k* is the maximum possible population size in particular environment or the carrying capacity; *r* is the intrinsic growth rate of the population and *t*-*gen* is doubling time or generation time of a population. **c** SCFAs production in microbial community co-culture with SCK6 and BsS-RS06550, respectively, including acetic acid (AA), propanoic acid (PA), and butyric acid (BA). Data are represented as mean ± SD, n = 5 repeats for (**a**, **c**). *p value < 0.05, **p < 0.01 and ***p < 0.001

SCK6 has been demonstrated to be an ideal host due to its excellent protein expression and transformation capabilities [26, 27]. Based on whole genome sequencing data, there is only one BA biosynthetic pathway in *B. subtilis*, the BA kinase pathway [28]. In recent years, a new BA synthetic pathway, the butyryl CoA: acetic acid CoA transferase (BCoAT) pathway, has been identified in most BA-producing microbiota in the intestine [29]. To improve BA production, we inserted the gene encoding of BCoAT into the genome of SCK6 to express the new BA synthesis pathway. Considering that bacterial biomass may also be an enhancement factor for BA production, the gene *skfA*, encoding sporulation killing factor, was disrupted to increase the growth rate [30]. Compared to SCK6, BsS-RS06550 produced a significantly greater amount of BA, approximately 3.80-fold higher (Fig. 1a, 0.238 ± 0.014 g/L vs 0.984 ± 0.027 g/L, *p *< 0.001). In addition, the growth rate of BsS-RS06550 was higher compared to that observed for SCK6 (Fig. 1b). The carrying capacity (*k*) (1.35 ± 0.007 vs 1.49 ± 0.021, *p *< 0.001) and a doubling time (*t*-*gen*) (0.451 ± 0.010 vs 0.630 ± 0.022, *p *< 0.001) of BsS-RS06550 were higher than that of SCK6, and a lower intrinsic growth rate (*r*) (1.54 ± 0.035 vs 1.10 ± 0.039, *p *< 0.001) was observed for BsS-RS06550. Furthermore, we simulated the intestinal environment in vitro to evaluate the BA productivity of BsS-RS06550 cultured with the gut microbial community [31, 32]. In the microbial community inoculated with BsS-RS06550, higher BA accumulation occurred compared to that observed for SCK6 (Fig. 1c, 0.095 ± 0.004 g/L vs 0.111 ± 0.009 g/L, *p *< 0.01). Acetic acid (AA) production in microbial community inoculated BsS-RS06550 was significantly decreased (0.165 ± 0.006 g/L vs 0.130 ± 0.011 g/L, *p *< 0.001), indicating that AA is a substrate for BA synthesis [33].

### Beneficial effects- of BsS-RS06550 on HFD-induced obesity in mice

**Fig. 2:**
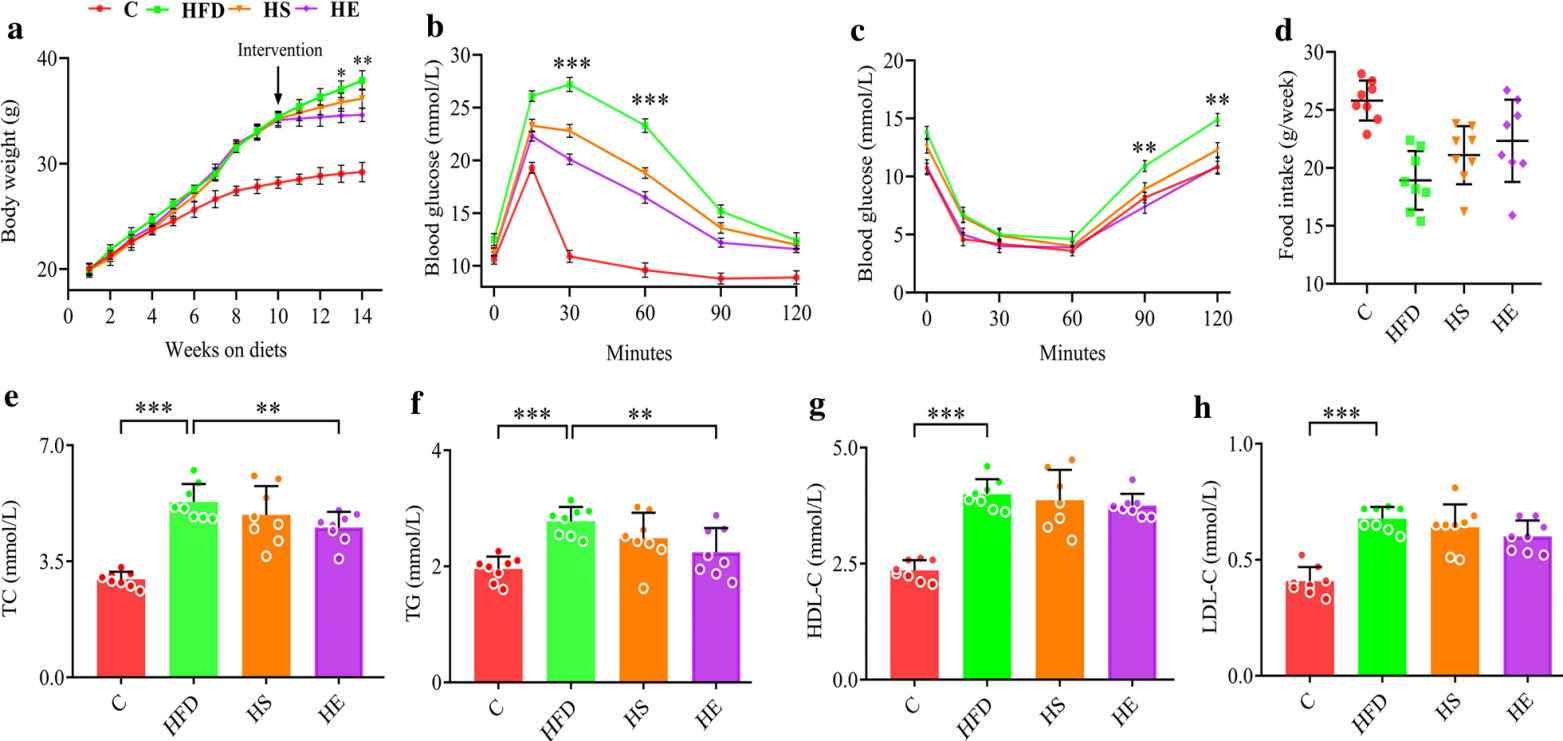
Effects of BsS-RS06550 on physiology and lipid metabolism in blood. **a** Weekly body weight, **b** glucose tolerance test following intervention. **c** Insulin tests following intervention, **d** weekly food intake, lipid metabolism in blood, TC (**e**), TG (**f**), HDL-C (**g**) and LDL-C (**h**). Data represented as mean ± SD, n = 8 mice/group for (**a**–**h**). *p value < 0.05, **p < 0.01 and ***p < 0.001

The experimental design included 4 groups (n = 8 for each group): a normal diet (C) group, a high-fat diet (HFD) group, an HFD + *Bacillus subtilis* SCK6 (HS) group and an HFD + BsS-RS06550 (HE). As shown in Fig. 2a, the body weights of mice in the HFD, HS and HE groups showed no differences before intervention, while BsS-RS06550 supplementation significantly decreased the body weight gain in the HFD-induced mice (HE vs HFD at 14 weeks, 34.60 ± 0.63 g vs 37.90 ± 0.88 g, *p *< 0.01). After 14 weeks, as shown in Fig. 2b, c, there were no significant differences in fasting glucose and insulin between all groups at the start of glucose tolerance test (GTT) and insulin tolerance test (ITT). BA has been proven to improve insulin response and regulate blood glucose [34]. Interestingly, the fasting glucose levels were significantly lower in the HE group compared with the HFD group (at 30 and 60 min, 20.1 ± 0.500 mmol/L vs 27.3 ± 0.656 mmol/L, 16.5 ± 0.751 mmol/L vs 23.3 ± 0.500 mmol/L, *p *< 0.001), and insulin levels in the HE group were significantly lower compared with those observed in the HFD group (at the end of ITT, 11.2 ± 0.31 mmol/L vs 14.7 ± 0.60 mmol/L, *p *< 0.01). Compared to the C mice, food intake was reduced in the HFD and HE groups (Fig. 2d).

### Serum analysis and liver injury

**Fig. 3:**
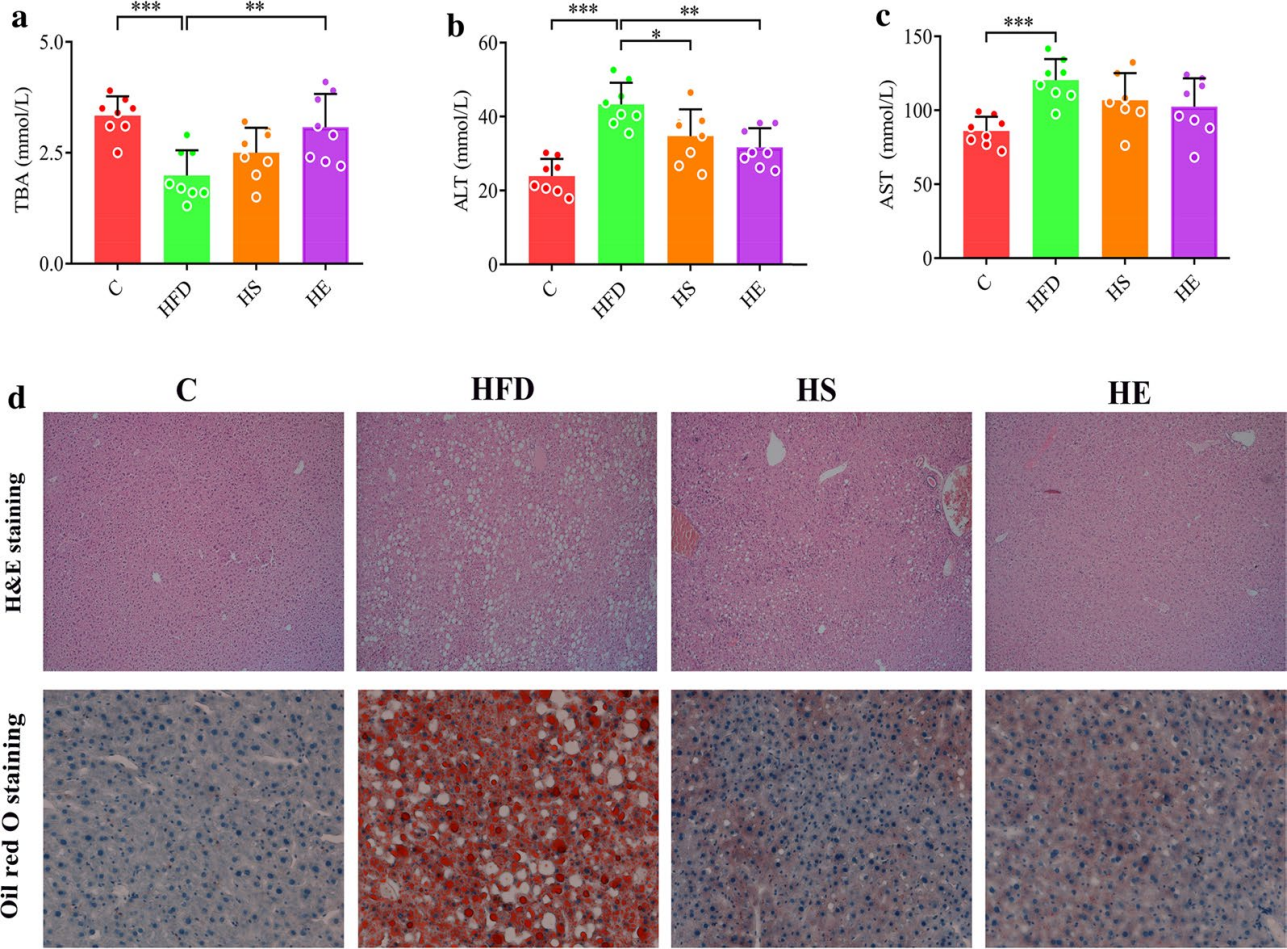
Effects of BsS-RS06550 on HFD-induced hepatic steatosis. **a** Serum TBA level, **b** serum ALT level, **c** serum AST level, **d** hematoxylin and eosin (H&E) staining and oil red O staining of and livers (×200). Data represented as mean ± SD, and n = 8 mice/group for (a–c). *p value < 0.05, **p < 0.01 and ***p < 0.001

Studies have shown that BA regulates serum biochemical indicators in HFD fed mice [35, 36]. The high-fat diet significantly increased the total cholesterol (TC), triglyceride (TG), high density lipoprotein (HDL), and low-density lipoprotein (LDL) levels in HFD group compared with those observed in the C group (Fig. 2, 5.30 ± 0.542 mmol/L vs 2.96 ± 0.224 mmol/L, 2.78 ± 0.249 mmol/L vs 1.95 ± 0.216 mmol/L, 4.00 ± 0.322 mmol/L vs 2.36 ± 0.219 mmol/L, 0.678 ± 0.051 mmol/L vs 0.408 ± 0.061 mmol/L, *p *< 0.001). Supplementation with BsS-RS06550 significantly lowered the TC and TG levels in the HE group compared with that observed in the HFD group (Fig. 2e, f, 4.53 ± 0.469 mmol/L vs 5.30 ± 0.542 mmol/L, 2.24 ± 0.419 mmol/L vs 2.78 ± 0.249 mmol/L, *p *< 0.01), and had no effect on HDL or LDL cholesterol levels (Fig. 2g, h). The level of total bile acids (TBAs) in serum significantly increased after the BsS-RS06550 intervention (Fig. 3a, HE vs HFD, 3.08 ± 0.754 mmol/L vs 1.99 ± 0.567 mmol/L, *p* < 0.01). As shown in Fig. 3b, the level of alanine aminotransferase (ALT) in the HFD group was higher than that observed in the HS (43.3 ± 5.87 mmol/L vs 34.7 ± 7.25 mmol/L, *p *< 0.05) and HE groups (43.3 ± 5.87 mmol/L vs 31.7 ± 5.18 mmol/L, *p *< 0.01). In contrast, no significant changes in aspartate aminotransferase (AST) levels were observed between the HFD and HS or HE groups (Fig. 3c). Significant liver injury was further assessed through histological analysis (Fig. 3d). Obvious macrovascular steatosis and fat accumulation were observed in hepatic cells of mice in the HFD group, whereas BsS-RS06550 supplementation attenuated hepatic steatosis and fat accumulation.

### Metabolic response of HFD-induced obesity in mice to BsS-RS06550

**Fig. 4:**
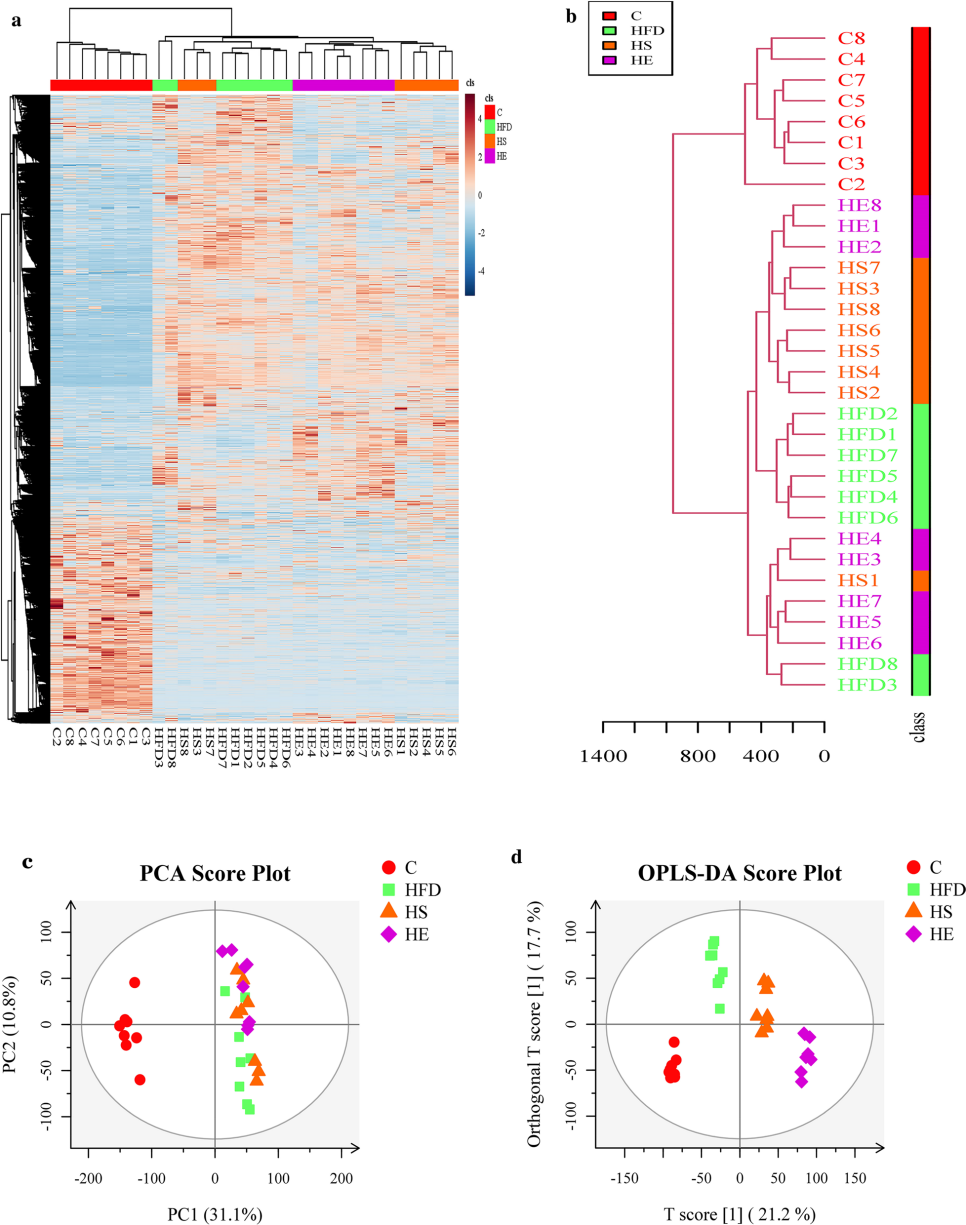
Untargeted fecal metabolomics analysis. **a** Hierarchical clustering of differentially metabolites in all groups. **b** Tree analysis of samples. **c** PCA score plots of fecal metabolic profiles, normal diet group (C), a high-fat diet group (HFD), HFD + *Bacillus subtilis* SCK6 group (HS) and HFD + BsS-RE06550 (HE). **d** OPLS-DA score plots of fecal metabolic profiling of C, HFD, HS and HE

Increasing evidence has shown that BA participates in energy and material metabolism [37], and the physiological alterations noted above may have been caused by differences in metabolism. In the present study, we collected fecal samples from all groups to assess metabolic changes. A total of 9726 potential biomarkers were filtered, and significant changes were observed among all groups (Fig. 4a, b). PCA and orthogonal OPLS-DA were performed on the LC–MS data and provided a general overview of clustering information (Fig. 4c, d). In the PCA model, there was no significant separation between HFD, HS and HE groups and all groups were separated from the C group. However, the OPLS-DA model, a supervised clustering method was compared to PCA, which provided greater discrimination power. The score plot showed that all groups were clearly separated from each other, and these results indicated that BsS-RS06550 altered the metabolic profiles of feces from mice in the HFD group.

### Metabolites and metabolic pathway analysis

**Fig. 5:**
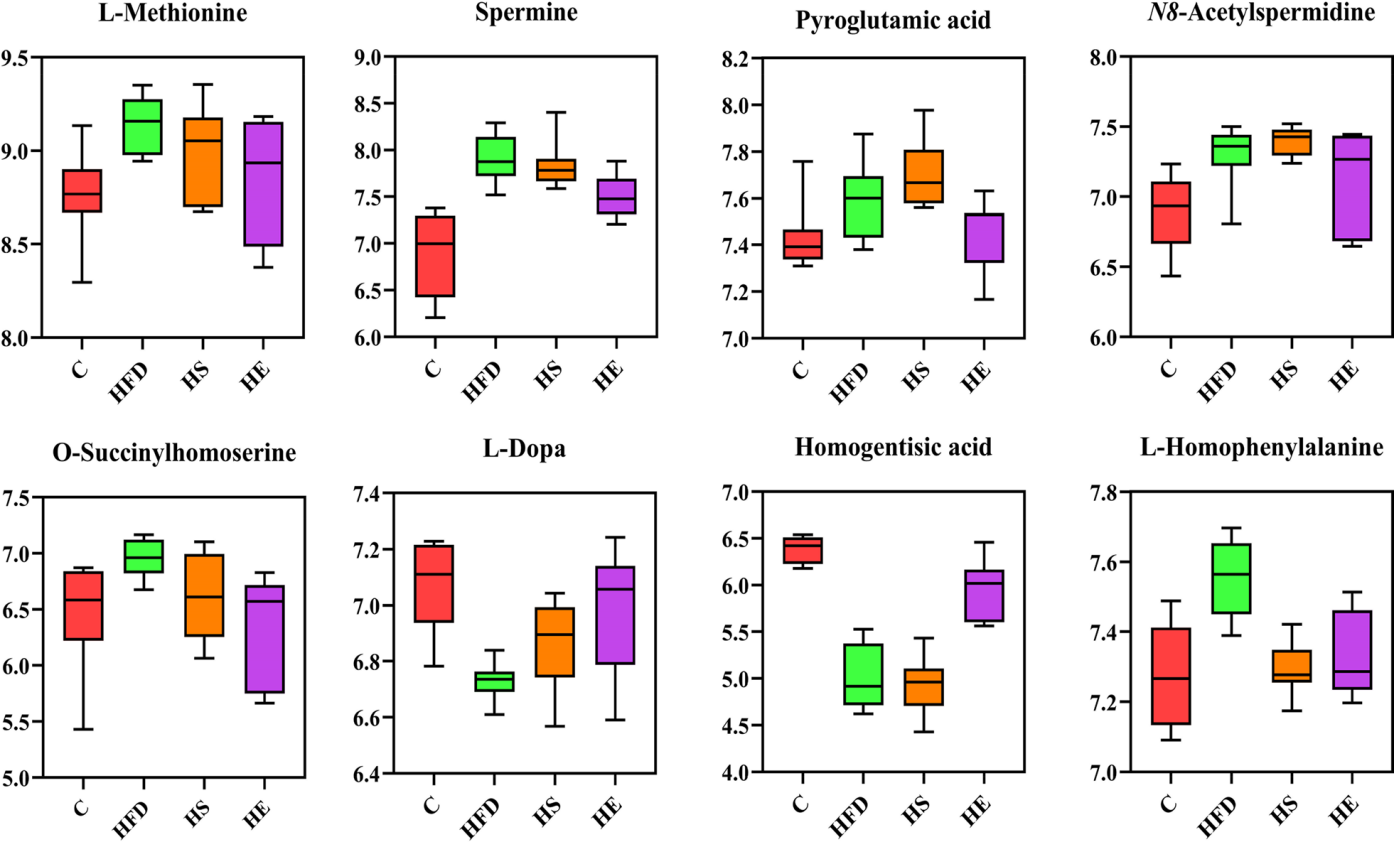
Box plots of relative abundance of significance metabolites in glutathione, methionine, tyrosine, phenylalanine metabolism, and C (control), HFD (high-fat diet), HFD + *Bacillus subtilis* SCK6 group (HS) and HFD + BsS-RS06550 (HE) groups (n = 8 for each group). Normalizing the intensity data with log function conversion (based on 10)

**Fig. 6:**
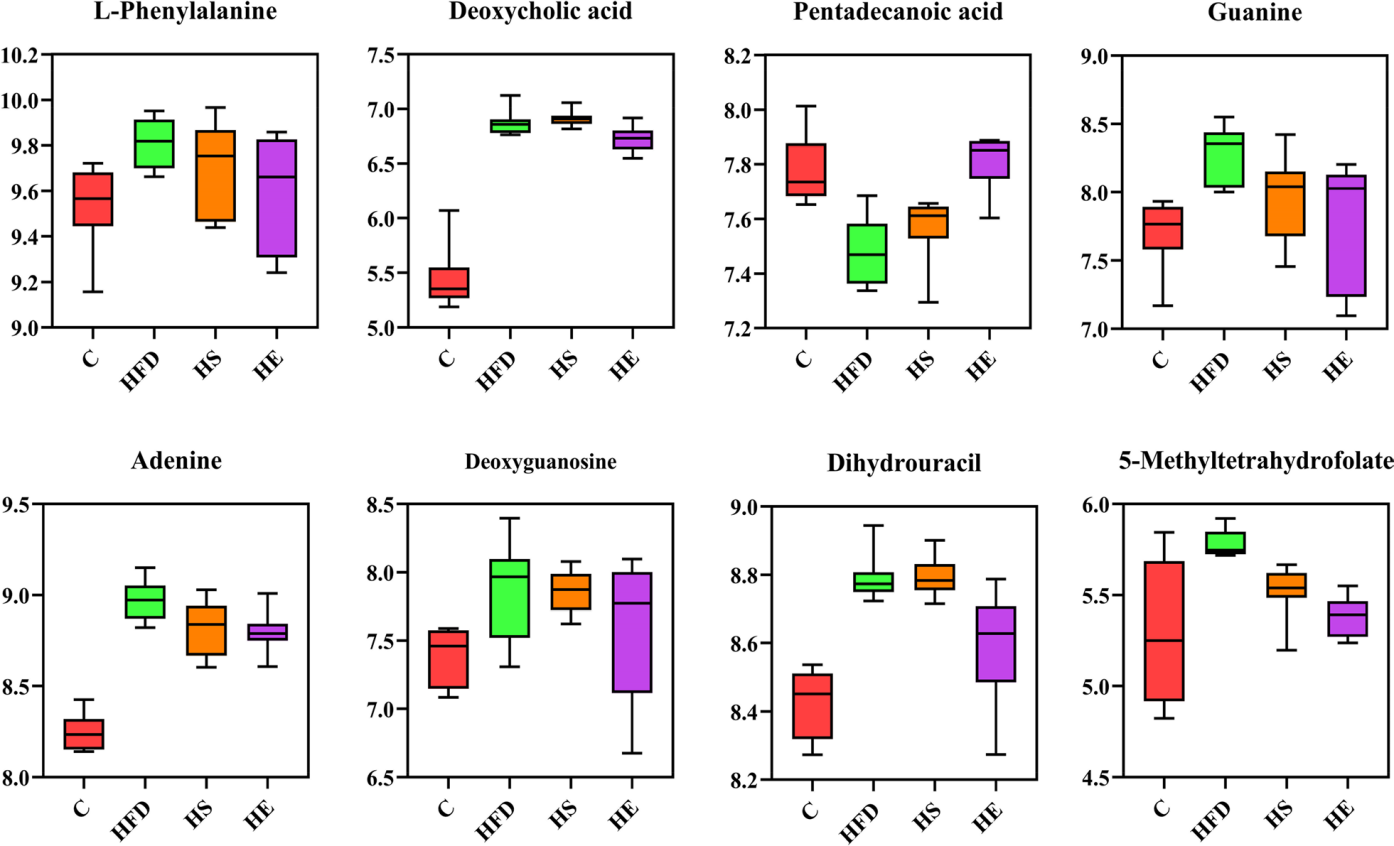
Box plots of relative abundance of significance metabolites in purine metabolism and secondary bile acid biosynthesis, and C (control), HFD (high-fat diet), HFD + *Bacillus subtilis* SCK6 group (HS) and HFD + BsS-RS06550 (HE) groups (n = 8 for each group). Normalizing the intensity data with log function conversion (based on 10)

Based on metabolomics analyses, researchers have adopted intervention approaches for the treatment of obesity [38]. The consumption of an HFD can increase energy extraction and decrease the production of obesity-suppressing SCFAs, resulting in obesity and metabolic disorders [39]. In addition, negative correlations have been shown to occur between BA and some metabol–ites in multiple metabolic pathways. The levels of metabolites significantly changed according to the OPLS-DA (VIP score > 1) and one way ANOVA (*p* value ≤ 0.05) results. All identified metabolites were calculated by high-accuracy quasi-molecular ion mass spectrometry with a mass error of < 20 ppm and classified by their metabolic functions and pathways. One hundred eight metabolites were identified as exhibiting significant differences and were semi-quantified on the basis of their exact mass, retention time and the comparison results with the KEGG database in all groups. The significant metabolites among the HFD, HS and HE groups are listed in Table 1. The levels of l-methionine, spermine, pyroglutamic acid, O-succinylhomoserine, l-homophenylalanine, l-phenylalanine, guanine, adenine, dihydrouracil, and 5-methyltetrahydrofolate were all significantly decreased in the HE group compared to those observed in the HFD group (Figs. 5, 6, *p *< 0.05). L-Dopa, homogentisic acid and pentadecanoic acid levels were significantly increased (*p *< 0.05) in the HFD group compared to those observed in the HE group, and only a lower content of L-homophenylalanine was observed in the HS group compared to that observed in the HFD group (Fig. 5, *p *< 0.05).

**Table 1:** Significance metabolites in fecal samples of HFD, HS and HE

Identification	Pathway	Content level	Significance
l-Methionine	Methionine metabolism	HFD > HE	9.14 ± 0.151 vs 8.84 ± 0.236*
Spermine	Glutathione metabolism	HFD > HE	7.90 ± 0.254 vs 7.51 ± 0.228**
Pyroglutamic acid	Glutathione metabolism	HFD > HE	7.61 ± 0.164 vs 7.37 ± 0.200**
*N8*-Acetylspermidine		HFD > HE	7.39 ± 0.219 vs 7.18 ± 0.272*
*O*-Succinylhomoserine	Methionine metabolism	HFD > HE	6.95 ± 0.168 vs 6.32 ± 0.4928*
l-Dopa	Tyrosine metabolism	HFD < HE	6.73 ± 0.066 vs 6.99 ± 0.222**
Homogentisic acid	Tyrosine metabolism	HFD < HE	5.00 ± 0.350 vs 5.94 ± 0.320***
l-Homophenylalanine	Phenylalanine, tyrosine and tryptophan biosynthesis	HFD > HE HFD > HS	7.55 ± 0.108 vs 7.33 ± 0.122** 7.55 ± 0.108 vs 7.29 ± 0.075^##^
l-Phenylalanine	Phenylalanine metabolism	HFD > HE	9.81 ± 0.108 vs 9.59 ± 0.251*
Deoxycholic acid	Secondary bile acid biosynthesis	HFD > HE	6.87 ± 0.117 vs 6.72 ± 0.118*
Pentadecanoic acid		HFD < HE	7.48 ± 0.124 vs 7.81 ± 0.102***
Guanine	Purine metabolism	HFD > HE	8.27 ± 0.216 vs 7.97 ± 0.469*
Adenine	Purine metabolism	HFD > HE	8.97 ± 0.110 vs 8.88 ± 0.114**
Deoxyguanosine	Purine metabolism	HFD > HE	7.86 ± 0.264 vs 7.64 ± 0.416*
Dihydrouracil	Pyrimidine metabolism	HFD > HE	8.79 ± 0.111 vs 8.59 ± 0.263**
5-Methyltetrahydrofolate	One carbon pool by folate, carbon fixation pathways in prokaryotes	HFD > HE	5.78 ± 0.075 vs 5.38 ± 0.108**

^*^HFD vs HE; ^#^HFD vs HS. */^#^*p* value < 0.05, **/^##^*p* < 0.01 and ***/^###^*p* < 001

**Fig. 7:**
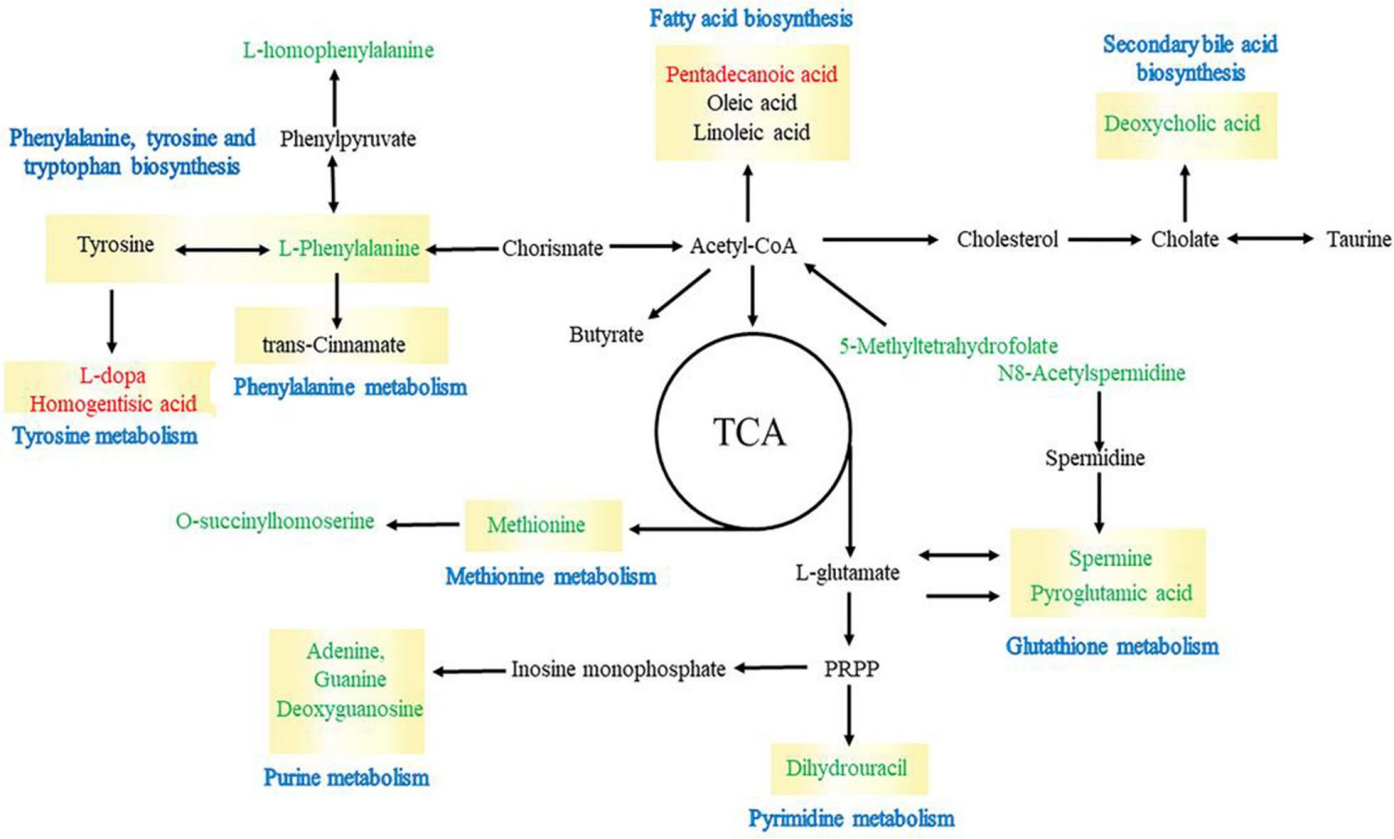
Schematic diagram of proposed metabolic pathways in all fecal samples. Red and green represent up- and downregulated metabolites, respectively

Up- and downregulation of metabolites herald changes in the metabolic pathway. In this study, we adopted fecal samples as the object to investigate the changes in metabolic pathways. Based on the significant difference metabolites, a network diagraph was constructed based on the KEGG pathway and literature for describing these potential characteristic metabolites and their metabolism (Fig. 7). The identified metabolites were mainly involved in methionine, purine, glutathione, tyrosine and cysteine metabolism, and biosynthesis of phenylalanine and secondary bile acid.

## Discussion

Obesity is a chronic disease characterized by long-term metabolic disorders and increases the risk of other diseases [1]. Microbiota-derived BA decreases the weights of mice with HFD induced obesity by weakening the activity of epithelial HDAC3 [40]. The results of previous studies have indicated that BA can improve metabolism by reducing energy intake and enhancing fat oxidation. Oral butyrate can suppress the activity of orexigenic neurons and reduce food intake, and it also prevents hypertriglyceridemia and hepatic steatosis. These observations suggest that BA has the potential for preventing or mitigating HFD-induced obesity by altering metabolism. Modified bacteria have been used to inhibit HFD-induced obesity in mice by producing NAPEs [14]. In this study, we genetically modified SCK6 to have enhanced BA production. The gene encoding BCoAT was knocked into SCK6 to introduce a new BA synthesis pathway, and the gene *skfA* was disrupted to increase biomass [30]. As expected, the modified BsS-RS06550 strain exhibited higher BA production and growth rate compared to that observed in the SCK6 strain (Fig. 1a, b, 0.238 ± 0.014 g/L vs 0.984 ± 0.027 g/L, *p* < 0.001). In addition, the BsS-RS06550 strain showed an increased capability for BA production in vitro co-culture environment compared to that observed for the SCK6 strain (Fig. 1c, 0.095 ± 0.004 g/L vs 0.111 ± 0.009 g/L, *p* < 0.01).

First, we evaluated the effect of BsS-RS06550 intervention in mice fed an HFD. The body weight gains of the mice fed BsS-RS06550 was lower in compared to that observed in the HFD mice (*p *< 0.01 at week 16). Furthermore, the glucose and insulin levels were improved by 4 weeks of the BsS-RS06550 treatment, BsS-RS06550 significantly decreased the levels of TC, TG and ALT in the HFD-fed mice. Moreover, BsS-RS06550 increased TBA levels and attenuated hepatic steatosis and fat accumulation. These results are consistent with those of other studies showing that the enhancement of BA regulates serum chemical indictors in mice with HFD-induced obesity [37, 41]. These results indicated that the use of BsS-RS06550 is a potential strategy for attenuating obesity in mice resulting from an HFD.

Fecal metabolomics has been focused on revealing changes in metabolites and metabolic pathways in diseases [36, 42, 43]. Thus, we performed metabolomics analysis of fecal samples to investigate changes in metabolite levels and metabolic pathways. The metabolites exhibiting significant changes are shown in Table 1, and sixteen metabolites were identified exhibiting different levels in the HS and HE groups compared to those observed in the HFD group. The changes of l-methionine, spermine and pyroglutamic acid reflected alterations in glutathione metabolism with the BsS-RS06550 treatment. Methionine is a sulfur-containing amino acid that is necessary for humans and animals and is involved in carbon metabolism and sulfur transfer [44]. Spermine is a metabolite of methionine that regulates the synthesis of nucleic acids and proteins in vivo, and *N*8-acetylspermidine is a polyamine present in living organisms that appears to be deacetylated to spermidine [45]. These studies have shown that the excessive intake of methionine increases fat accumulation and weight gain, increasing the risk of obesity and dyslipidemia. We observed the downregulation of l-methionine (*p *< 0.05) and spermine (*p *< 0.01) levels in the HE group compared with those observed in the HFD group, indicating that BsS-RS06550 alleviated adiposis in mice fed an HFD (Fig. 5). In addition, as an intermediate product of methionine synthesis, *O*-succinylhomoserine level was significantly decreased in the HE group compared with those observed in the HFD group (*p *< 0.01). Interestingly, both l-methionine and *O*-succinylhomoserine are associated with methionine metabolism. Pyroglutamic acid is a circulating amino acid in animals and plants, that is commonly used as an intermediate product in the process of amino acid metabolism and enzymatic reactions. Furthermore, the accumulation of pyroglutamic acid results in nonalcoholic steatohepatitis [46]. These findings are consistent with our results shown in Fig. 5, indicating that the BsS-RS06550 treatment decreased the levels of l-methionine, spermine, pyroglutamic acid and *O*-succinylhomoserine and improved disorders associated with glutathione and methionine metabolism.

l-Dopa and homogentisic acid are metabolites involved in tyrosine metabolism. l-Dopa is the precursor of the neurotransmitter dopamine, and its use is a potential strategy for controlling weight after chronic bilateral subthalamic stimulation surgery [47]. Homogentisic acid is another metabolite involved in tyrosine metabolism. A study by Nguyen reported that homogentisic acid is an effective α-glucosidase inhibitor for improving glucose levels in blood [48]. In this study, the levels of l-Dopa (*p *< 0.01) and homogentisic acid (*p *< 0.001) were significantly increased in the HE group compared with that observed in the HFD group (Fig. 5), and the effects of l-Dopa and homogentisic acid were confirmed in our study (Fig. 2b, c). l-Homophenylalanine and l-phenylalanine are involved in the metabolism and biosynthesis of phenylalanine. These amino acids can be oxidized to tyrosine and are involved in the synthesis of neurotransmitters and hormones. In addition, phenylalanine has been identified as a potential biomarker in obesity [49]. The glucose and lipid metabolism disorders induced by an HFD may be alleviated by BsS-RS06550 via decreased the levels of phenylalanine (Fig. 6). Thus, these results suggest that BsS-RS06550 can impact the metabolism of tyrosine and phenylalanine.

Deoxycholic acid (DCA) is bile acid that showed significantly different levels between the HE and HFD groups (Fig. 6), and the BsS-RS06550 treatment significantly decreased the level of DCA in mice in the HE group compared to that observed in the HFD group (*p *< 0.05). High levels of DCA results in adverse health effects, which was confirmed in mice fed an HFD [50]. Pentadecanoic acid is a marker of dairy fat consumption and type 2 diabetes, which is negatively correlated with obesity [51]. Consistent with our results, the pentadecanoic acid was significantly decreased in the HFD group compared to the C group (Fig. 6, *p *< 0.001), and was increased in the HE with BsS-RS06550 treatment compared with that observed in the HFD group (Fig. 6, *p *< 0.001).

In addition, guanine, adenine, deoxyguanosine and dihydrouracil are involved in purine metabolism, which has important effects on adiposity and insulin resistance [52–54]. Nucleotides have a variety of functions, including acting as synthetic materials for nucleic acids, external stimuli messengers, energy conversion (ATP and GTP) mediators, coenzymes and biochemical reaction regulators. Studies have shown that higher levels of nucleotides were detected in obese patients compared to normal individuals, consistent with our results (Fig. 6). The results of this study showed that the levels of guanine, adenine, deoxyguanosine and dihydrouracil were significantly decreased in the HE group compared with that observed in the HFD group (*p *< 0.05). Furthermore, 5-methyltetrahydrofolate is involved in carbon metabolism and provides materials for the synthesis of nucleotides. BsS-RS06550 treatment decreased the level of 5-methyltetrahydrofolate in the HE group compared to that observed in the HFD group (Fig. 6, *p *< 0.05). Based on our results, the regulation of purine metabolism is also a pathway through which BsS-RS06550 could attenuate adiposity in mice fed an HFD.

## Conclusion

In summary, the results of our study provide a potential strategy for the treatment of obesity. The genetically modified *Bacillus subtilis* SCK6 strain BsS-RS06550 showed beneficial effects toward obesity, fasting blood glucose, insulin resistance, hepatic steatosis and fat accumulation. Furthermore, a fecal metabolomics approach based on nontargeted LC-MS was used to investigate metabolic alterations resulting from the BsS-RS06550 treatment. Sixteen metabolites showing potential significant difference were observed between HFD and HE groups, including l-methionine, spermine, pyroglutamic acid, *N*8-acetylspermidine, *O*-succinylhomoserine, l-dopa, homogentisic acid, l-homophenylalanine, l-phenylalanine, DCA, pentadecanoic acid, guanine, adenine, deoxyguanosine, dihydrouracil and 5-methyltetrahydrofolate. BsS-RS06550 improved the levels of metabolites and pathways involved in metabolic disorders in mice fed a high-fat diet, including glutathione, methionine, tyrosine, phenylalanine, purine metabolism and secondary bile acid biosynthesis. These results reveal that the metabolism of nucleotides and amino acids plays a key role in obesity induced by a high-fat diet, and the use of BsS-RS06550 is a potentially and effective strategy for alleviating obesity and metabolic disorders.

## Methods

### Strains, media and growth conditions

The bacterial strains and plasmids used in this study are listed Additional file 1: Table S1. *Escherichia coli* DH5α was used for DNA cloning and plasmid construction. *Bacillus subtilis* SCK6 was used as the parent strain for strain constructions, and transformed with the Spizizen’s minimal medium. All strains were routinely cultured in Luria-Bertani (LB) liquid medium and LB agar plates at 37 °C. Kanamycin was used to select pCas at a final concentration of 100 μg/mL, and Spectinomycin was used at 100 μg/mL to select pTargetF plasmids.

### BsS-RS06550 construction and in vitro experiments

A two-plasmid system containing a genome editing system was constructed for engineering *B. subtilis* SCK6, including pCas and pTargetF [55, 56]. pCas provides protein cas9 to cut the region of interest, and pTargetF harbors gRNA for targeting the editing region to be edited. A new gRNA scaffold was cloned into pTargetF for constitutive sgRNA expression. The gRNA contains three parts, a modified promoter P_43_, guide-RNA and donor fragments for repairing double-strand breaks. One insertion and one deletion were performed to construct BsS-RS06550. The gene encoding the coding BCoAT was inserted into the genome of SCK6 to introduce a new BA synthesis pathway, and this insertion also disrupted the gene *sdpC*. In addition, the gene encoding *skfA* was disrupted to improve biomass. The primers *skfA*-for and *skfA*-rev, *sdpC*-for and *sdpC*-rev, B-CoA: A CoA-for and B-CoA: A CoA-rev were used to verify deletion of the genes *skfA* and *sdpC*, and the insertion of BCoAT. The primers *skfA*-1-for and *skfA*-1-rev, and *sdpC*-1-for and *spdC*-1-rev were used for verification of homologous recombination. A spectinomycin resistance gene allows for the selection of positive monoclonal isolates of different engineered *B. subtilis* SCK6 strains. All primers and N_20_ sequences used in this study relisted Additional file 1: Table S2. Growth curves were used to analyze microbial growth rates [57]. The microbial community was collected from fresh fecal samples from mice fed a high-fat diet and co-cultured with BsS-RS06550 in vitro. The culturing process was performed as described in a previous study [31, 32], and bacterial supernatants were collected by centrifugation to determine SCFAs profiles.

### SCFAs analysis

The YCFA medium supernatants were collected by centrifugation after 24 h of cultivation at 37 °C. The concentrations of SCFAs were measured by gas chromatography–mass spectrometry (GC–MS). First, 0.4 mL of 50% (*v/v*) sulfuric acid and 2 mL diethyl ether were added to 2 mL of supernatants for acidification and enrichment of SCFAs. The supernatants were collected after centrifuging at 4 °C, centrifuged at 3000 r/min for 5 min and then filtered through 0.22-μm nylon filters. Calcium chloride was used to remove water, and the supernatants were used for GC–MS detection. The detailed GC–MS process is shown in the Additional file 1.

### Animals and treatment regime

32 male C57 BL/6 J mice (3-5 weeks old) were housed and maintained under constant temperature (22 ± 3 °C) and humidity (55 ± 10%). The mice had free access to water and food and were subjected to 12 h light–dark cycles during one-week acclimation. The 32 mice were randomly distributed in four groups (n = 8 per group): control group (C), high-fat diet (HFD) group, HFD + *Bacillus subtilis* SCK6 (HS) group and HFD + BsS-RS06550 (HE) group. The mice in HFD, HS and HE were given by gavage once a day with 200 μL of control ddH_2_O, SCK6 (10^8^ CFU/mL) and BsS-RS06550 (10^8^ CFU/mL), respectively. All treatments were given for 4 weeks, and the mice fasted overnight before being sacrificed at the conclusion of the study. Blood, liver and gut tissues were collected from the sacrificed animals and being stored at − 80 °C. Body weight and food intake were measured once per week for each mouse.

### Liver histology and serum analysis

Liver tissues were fixed with 4% formalin and processed for paraffin embedding. Tissue sections were cut into 5-μm thick slices and stained with hematoxylin and eosin (H&E) using a standard procedure. The tissue samples were observed and photographed with a microscope CX31 (Olympus).

Liver tissues fixed with 10% formalin were rapidly frozen with liquid nitrogen and then sectioned into 5-μm thick slices. The sectioned liver tissue slides were placed in absolute propylene glycol for 5 min to avoid carrying water over into the Oil Red O. The slides were stained with pre-warmed Oil Red O solution for 30 min at room temperature, and then Stained in Mayer’s hematoxylin solution for 30 s. After staining, the slides were washed three times with water and then observed under a microscope Zeiss Obverse 7 (Zeiss).

Blood samples were drawn after a 12-h fast. Serum/plasma was separated before being stored at − 80 °C. Serum biochemical parameters were measured using a Mindray BS-2000M instrument (Shenzhen, China) according to the manufacturer’s guidelines. For insulin tolerance tests (ITTs), mice received an intraperitoneal injection of human insulin in an ad libitum-fed state after an overnight fasting. For glucose tolerance tests (GTTs), mice received an oral administration of glucose after an overnight fasting. Blood glucose levels were measured using a Roche ACCU-CHEK active blood glucose meter (Basel, Switzerland) at the indicated time points. Plasma free fatty acid levels were measured using a quantification kit (WAKO).

### Nontargeted LC–MS for measurements of metabolites in fecal samples

Fecal samples (100 mg) and 500 μL ddH_2_O (4 °C) were transferred into 2 mL centrifuge tubes and vortexed for 60 s. Subsequently, 1000 μL of methanol (pre-cooled at − 20 °C) was transferred into the tubes and vortexed for 30 s. Then, the solutions were sonicated for 10 min at room temperature and then incubated for 30 min on the ice. Supernatant samples were collected after centrifugation for 10 min at 14, 000 rpm and 4 °C. The supernatant samples were blow-dried by vacuum concentration and then dissolved in 400 μL of a 2-chlorobenzalamine (4 ppm) methanol aqueous solution (1:1, 4 °C). After 0.22-μm membrane filtration, the samples were ready for LC–MS detection. The specific untargeted LC–MS conditions are shown in supplementary materials.

### Statistical analysis

In vitro BA production data were analyzed in triplicate. Data were expressed as the mean ± standard deviation (SD) and were subjected to one-way analysis of variance (ANOVA) with Turkey’ test and graphics presentation using GraphPad Prism software 8.0 (La Jolla, CA). The nontargeted LC–MS raw data were processed by Proteowizard software (v3.0.8789). Afterwards, peaks identification, peaks filtration and peaks alignment were administrated by R package XCMS (v3.3.2), and the data matrix was established based on mass to charge ratio (m/z), retention time (rt) and intensity. Principal component analysis (PCA) and orthogonal partial least squares discriminant analysis (OPLS-DA) were administrated with the data matrix using R package ropls. GraphPad Prism software 8.0 for ANOVA was used for statistical analyses between groups. *p < 0.05, **p < 0.01 and ***p < 0.001. Metabolic pathway and metabolites were identified with databases, HMDB (http://www.hmdb.ca), Metalin (http://metlin.scripps.edu), massbank (http://www.massbank.jp/), LipidMaps (http://www.lipidmaps.org), mzclound (http://www.mzcloud.org) and KEGG (http://www.kegg.com).

## Supplementary information

**Additional file 1:** Additional methods and materials in this study.

## Data Availability

All data generated or analysed during this study are included in this published article.

## Abbreviations

AA: Acetic acid
PA: Propanoic acid
BA: Butyric acid
LC–MS: Liquid chromatograph–mass spectrometer
TG: Triglyceride
TC: Total cholesterol
HDL: High-density lipoprotein
LDL: Low-density lipoprotein
NAPEs: *N*-Acyl-phosphatidylethanolamines
SCFAs: Short-chain fatty acids
LB: Luria–Bertani
GC-MS: Gas chromatography–mass spectrometry
HFD: High-fat diet
ITTs: Insulin tolerance tests
GTTs: Glucose tolerance tests
SD: Standard deviation
PCA: Principal component analysis
OPLS-DA: Orthogonal partial least squares discriminant analysis
TBA: Total bile acids
ALT: Alanine aminotransferase
AST: Aspartate aminotransferase
DCA: Deoxycholic acid

## References

[1] Mulders RJ, de Git KCG, Schéle E, Dickson SL, Sanz Y, Adan RAH (2018). Microbiota in obesity: interactions with enteroendocrine, immune and central nervous systems. Obes Rev.

[2] Nyberg ST, Batty GD, Pentti J, Virtanen M, Alfredsson L, Fransson EI (2018). Obesity and loss of disease-free years O6 wing to major non-communicable diseases: a multigroup study. Lancet Public Health..

[3] Ng M, Fleming T, Robinson M, Thomson B, Graetz N, Margono C (2014). Global, regional, and national prevalence of overweight and obesity in children and adults during 1980–2013: a systematic analysis for the global burden of disease study 2013. Lancet.

[4] Batsis JA, Villareal DT (2018). Sarcopenic obesity in older adults: aetiology, epidemiology and treatment strategies. Nat Rev Endocrinol.

[5] Ford ES, Mokdad AH, Giles WH, Galuska DA, Serdula MK (2005). Geographic variation in the prevalence of obesity, diabetes, and obesity-related behaviors. Obes Res.

[6] Wan Y, Wang FL, Yuan JH, Li J, Jiang DD, Zhang JJ (2017). Effects of macronutrient distribution on eight and related cardiometabolic profile in healthy non-obese chinese: a 6-month, randomized controlled-feeding trial. EBioMedicine..

[7] Seconda L, Egnell M, Julia C, Touvier M, Hercberg S, Pointereau P (2019). Association between sustainable dietary patterns and body weight, overweight, and obesity risk in the nutriNet-Santé prospective cohort. Am J Clin Nutr.

[8] Rankin W, Wittert G (2015). Anti-obesity drugs. Curr Opin Lipidol.

[9] Kang JG, Park CY (2012). Anti-obesity drugs: a review about their effects and safety. Diabetes Metab J..

[10] Spittal MJ, Frühbeck G (2018). Bariatric surgery: many benefits, but emerging risks. Lancet Diabetes Endo..

[11] van Neerven RJJ, Savelkoul H (2017). Nutrition and allergic diseases. Nutrients..

[12] Li Q, Liu F, Liu J, Liao S, Zou Y (2019). Mulberry leaf polyphenols and fiber induce synergistic antiobesity and display a modulation effect on gut microbiota and metabolites. Nutrients..

[13] Imamura F, Fretts A, Marklund M, Ardisson Korat AV, Yang WS (2018). Fatty acid biomarkers of dairy fat consumption and incidence of type 2 diabetes: a pooled analysis of prospective group studies. Plos Med..

[14] Yoneshiro T, Wang Q, Tajima K, Matsushita M, Maki H, Igarashi K (2019). BCAA catabolism in brown fat controls energy homeostasis through SLC25A44. Nature.

[15] O’Grady J, O’Connor EM, Shanahan F (2019). Dietary fibre in the era of microbiome science. Aliment Pharm Ther..

[16] Ríos-Covián D, Ruas-Madiedo P, Margolles A, Gueimonde M, De los Reyes-Gavilán CG, Salazar N (2016). Intestinal short chain fatty acids and their link with diet and human health. Front Microbiol..

[17] Li Z, Yi CX, Katiraei S, Kooijman S, Zhou EC, Chung CK (2017). Butyrate reduces appetite and activates brown adipose tissue via the gut-brain neural circuit. Gut.

[18] Gao RY, Zhu CL, Li H, Yin MM, Pan C, Huang LS (2017). Dysbiosis signatures of gut microbiota along the sequence from healthy, young patients to those with overweight and obesity. Obesity..

[19] Takahashi M, Mccartney E, Knox A, Francesch M, Oka K, Wada K (2018). Effects of the butyric acid-producing strain *Clostridium butyricum* MIYAIRI 588 on broiler and piglet zootechnical performance and prevention of necrotic enteritis. Anim Sci J..

[20] Miquel S, Martı R, Chatel JM, Rossi O, Bermu LG, Sokol H (2013). *Faecalibacterium prausnitzii* and human intestinal health. Curr Opin Microbiol.

[21] Chen ZY, Guo LL, Zhang Y, Rosemary LW, Julie SP, Richard LP (2014). Incorporation of therapeutically modified bacteria into gut microbiota inhibits obesity. J Clin Invest..

[22] Isabella VM, Ha BN, Castillo MJ, Lubkowicz DJ, Rowe SE, Millet YA (2018). Development of a synthetic live bacterial therapeutic for the human metabolic disease phenylketonuria. Nat Biotechnol.

[23] He YY, Mao CX, Wen H, Chen ZY, Lai T, Li LG (2017). Influence of Ad libitum feeding of piglets with *Bacillus subtilis* fermented liquid feed on gut flora, luminal contents and health. Sci Rep..

[24] Ozdemir T, Fedorec AJH, Danino T, Barnes CP (2018). Synthetic biology and engineered live biotherapeutics: toward increasing system complexity. Cell Syst..

[25] Hansen NL, Miettinen K, Zhao Y, Ignea C, Andreadelli A, Raadam MH (2020). Integrating pathway elucidation with yeast engineering to produce polpunonic acid the precursor of the anti-obesity agent celastrol. Microb Cell Fact.

[26] Zhang XZ, Zhang YHP (2011). Simple, fast and high-efficiency transformation system for directed evolution of cellulase in *Bacillus subtilis*. Microb Biotechnol.

[27] Bashir S, Sadaf S, Ahmad S, Akhtar MW (2015). Enhanced and secretory expression of human granulocyte colony stimulating factor by *Bacillus subtilis* SCK6. Biomed Res Int.

[28] Kunst F, Ogasawara N, Moszer I, Albertini AM, Alloni G, Azevedo V (1997). The complete genome sequence of the gram-positive bacterium *Bacillus subtilis*. Nature.

[29] Louis P, Duncan SH, McCrae SI, Millar J, Jackson MS, Flint HJ (2004). Restricted distribution of the butyrate kinase pathway among butyrate-producing bacteria from the human colon. J Bacteriol.

[30] Liu WT, Yang YL, Xu YQ, Lamsa A, Haste NM, Yang JY (2010). Imaging mass spectrometry of intraspecies metabolic exchange revealed the cannibalistic factors of *Bacillus subtilis*. PNAS.

[31] De Paepe K, Verspreet J, Verbeke K, Raes J, Courtin CM, Van de Wiele T (2018). Introducing insoluble wheat bran as a gut microbiota niche in an in vitro dynamic gut model stimulates propionate and butyrate production and induces colon region specific shifts in the luminal and mucosal microbial community. Environ Microbiol.

[32] Di T, Chen GJ, Sun Y, Ou SY, Zeng XX, Hong Y (2018). In vitro digestion by saliva, simulated gastric and small intestinal juices and fermentation by human fecal microbiota of sulfated polysaccharides from gracilaria rubra. J Funct Foods..

[33] Merklein K, Fong SS, Deng Y (2014). Production of butyric acid by a cellulolytic actinobacterium thermobifida fusca on cellulose. Biochem Eng J.

[34] Muller NT, Zhang MY, Juraschek SP, Miller ER, Appel LJ (2020). Effects of high-fiber diets enriched with carbohydrate, protein, or unsaturated fat on circulating short chain fatty acids: results from the omniHert randomized trial. Am J Clin Nutr.

[35] Hyde PN, Sapper TN, Crabtree CD, LaFountain RA, Bowling ML, Buga A (2019). Dietary carbohydrate restriction improves metabolic syndrome independent of weight loss. JCI Insight..

[36] Liang YJ, Lin CL, Zhang YP, Deng YJ, Liu C, Yang Q (2018). Probiotic mixture of *Lactobacillus* and *Bifidobacterium* alleviates systemic adiposity and inflammation in non-alcoholic fatty liver disease rats through Gpr109a and the commensal metabolite butyrate. Inflammopharmacology.

[37] Koh A, De Vadder F, Kovatcheva-Datchary P (2016). From dietary fiber to host physiology: short-chain fatty acids as key bacterial metabolites. Cell.

[38] Saltiel AR (2016). New therapeutic approaches for the treatment of obesity. Sci Transl Med..

[39] Fang QY, Hu JL, Nie QX, Nie SP (2019). Effects of polysaccharides on glycometabolism based on gut microbiota alteration. Trends Food Sci Tech..

[40] Whitt J, Woo V, Lee P, Moncivaiz J, Haberman Y, Tso P (2018). Disruption of epithelial HDAC3 in intestine prevents diet-induced obesity in mice. Gastroenterology.

[41] Cirulli ET, Guo LL, Swisher CL, Shah N, Huang L, Napier LA (2019). Profound perturbation of the metabolome in obesity is associated with health risk. Cell Metab.

[42] Kuriz CB, Millet YA, Puurunen MK, Perreault M, Charbonneau MR, Isabell VM (2019). An engineered *E. coli* nissle improves hyperammonemia and survival in mice and shows dose-dependent exposure in healthy humans. Sci Transl Med..

[43] Wang L, Zeng B, Liu Z, Liao Z, Zhong Q, Gu L (2018). Green tea polyphenols modulate colonic microbiota diversity and lipid metabolism in high-fat diet treated HFA mice. J Food Sci.

[44] Orgeron ML, Stone KP, Wanders D, Cortez CC, Van NT, Gettys TW (2014). The impact of dietary methionine restriction on biomarkers of metabolic health. Pron Mol Biol Trans Sci..

[45] Mudumba S, Menezes A, Fries D, Blankenship J (2002). Differentiation of PC12 cells induced by *N*8-acetylspermidine and by *N*8-acetylspermidine deacetylase inhibition. Biochem Pharmacol.

[46] Qi S, Xu D, Li Q, Xie N, Xia J, Huo Q (2017). Metabonomics screening of serum identifies pyroglutamate as a diagnostic biomarker for nonalcoholic steatohepatitis. Clin Chim Acta.

[47] Guimarães J, Matos E, Rosas MJ, Vieira-Coelho A, Borges N, Correia F (2009). Modulation of nutritional state in parkinsonian patients with bilateral subthalamic nucleus stimulation. J Neurol.

[48] Nguyen VB, Nguyen AD, Wang SL (2017). Utilization of fishery processing by-product squid pens for α-glucosidase inhibitors production by *Paenibacillus* sp. Mar Drugs..

[49] Kim DJ, Yoon S, Ji SC, Yang J, Kim YK, Lee SH (2018). Ursodeoxycholic acid improves liver function via phenylalanine/tyrosine pathway and microbiome remodelling in patients with liver dysfunction. Sci Rep..

[50] Lin A, An Y, Tang H, Wang Y (2019). Alterations of bile acids and gut microbiota in obesity induced by high fat diet in rat model. J Agr Food Chem..

[51] Zhou J, Tang LL, Shen CW, Wang J (2018). Green tea polyphenols modify gut-microbiota dependent metabolisms of energy, bile constituents and micronutrients in female sprague-dawley rats. J Nutr Biochem.

[52] Yang X, Zhao Y, Sun Q, Yang Y, Gao Y, Ge W (2019). Adenine nucleotiede-mediated regulation of hepatic PTP1B activity in mouse models of type 2 diabetes. Diabetologia.

[53] Zhang X, Chen Y, Zhu J, Zhang M, Ho CT, Huang Q (2018). Metagenomics analysis of gut microbiota in a high fat diet-induced obesity mouse model fed with (−)-epigallocatechin 3-*O*-(3-*O*-methyl) gallate (EGCG3). Mol Nutr Food Res.

[54] Xiao Y, Li X, Zeng X, Wang H, Mai Q, Cheng Y (2019). A low ω-6/ω-3 ratio high-fat diet improves rat metabolism via purine and tryptophan metabolism in the intestinal tract, while reversed by inulin. J Agric Food Chem..

[55] Jiang Y, Chen B, Duan C, Sun B, Yang J, Yang S (2015). Multigene editing in the *Escherichia coli* genome via the CRISPR-cas9 system. Appl Environ Microb..

[56] Zhang K, Duan X, Wu J (2016). Multigene disruption in undomesticated *Bacillus subtilis* ATCC 6051a using the CRISPR/cas9 system. Sci Rep..

[57] Sprouffske K, Wagner A (2016). Growthcurver: an R package for obtaining interpretable metrics from microbial growth curves. BMC Bioinf.

