# Supplementary material for: Engineered butyrate-producing bacteria prevents high fat diet-induced obesity in mice
Source: Microb Cell Fact. 2020 Apr 25;19:94. doi: 10.1186/s12934-020-01350-z (PMC7183672; doi:10.1186/s12934-020-01350-z)
Supplement: Supplementary file 1 — Additional file 1: Additional methods and materials in this study. [file 12934_2020_1350_MOESM1_ESM.docx]

**Additional material**

Engineered butyrate-producing bacteria prevents high-fat diet-induced obesity in mice

**Methods**

**Detecting microbial growth curves**

The BsS-RS06650 was inoculated from a freshly transformed single colony on LB agar plate into 5 mL medium, and cultivated at 37 °C until OD_600_ value reached 1. Then strains were transformed into 0.2 mL fresh liquid LB medium culturing for 24 h, and growth curves were measured by automatic growth curve analyser Bioscreen C MBR. R package growth curves was used for growth rate.

**GC-MS process**

Gas chromatographic-mass spectrometric (GC–MS) analysis were carried out using an 7890GC-5977MS equipped with a DB-WAX column (5% phenylmethylsiloxane) and a split injector (split 1:50, temperature 250 °C). Helium of 99.999% purity was used as a carrier gas. The electron impact source temperature was 230 °C with an electron energy of 70 eV. The quadrupole temperature was 150 °C and the interface between GC and MS temperature was 250 °C. MS temperature process: initial 60 ℃, maintain 2 minutes, increase to 220 ℃ at 10 ℃/minute, maintain 20 minutes.

**Nontargeted LC-MS**

Chromatographic separation was accomplished in an Thermo Ultimate 3000 system equipped with an ACQUITY UPLC®HSS T3 (150 × 2.1 mm, 1.8 μm, Waters) column maintained at 40 ℃. The temperature of the autosampler was 8 ℃. Gradient elution of analytes was carried out with 0.1% formic acid in water (C), and 0.1% formic acid in acetonitrile (D) or 5 mM ammonium format in water (A) and acetonitrile (B) at a flow rate of 0.25 mL/minutes. Injection of 2 μL of each sample was done after equilibration. An increasing linear gradient of solvent B (v/v) was used as follows: 0~1 minutes, 2% B/D; 1~9 minutes, 2%~50% B/D; 9~12 minutes, 50%~98% B/D; 12~13.5 min, 98% B/D; 13.5~14 minutes, 98%~2% B/D; 14~20 min, 2% D-positive model (14~17 minutes, 2% B-negative model).

**Table S1 Strains and plasmids used in this study**

| Strain or plasmid | Characteristics | Source |
| --- | --- | --- |
| **Strains** |  | |
| *E. coli* |  |  |
| Trans-DH5α | F-φ80 lac ZΔM15 *Δ*(lacZYA-arg F) U169 endA1 recA1 hsdR17(rk-,mk+) supE44λ- thi -1 gyrA96 relA1 phoA | TransGen Biotech |
| *B. subtilis* |  |  |
| SCK6 | ErmR, 1A751 derivate, *lacA*::P*xylA*-*comK* | [26] |
| RS06550 | ErmR, 1A751 derivate, *lacA*::P*xylA*-*comKΔskfA ΔsdpC* :: butyryl-CoA: acetate: CoA transferase | This work |
| ***Plasmids*** |  | |
| pCas | *repA*101(Ts) *kan* *P_cas_*-cas9 *P_araB_*-*Red lacl^q^* *P_trc_-sgRNA-pMB1* | [58] |
| pTargetF | Harboring sgRNAs, with or without donor DNAs | [59] |
| pTargetF-P1 | *pMB1 aadA* sgRNA-*skfA* | This work |
| PTargetF-P2 | *pMB1 aadA* sgRNA-*sdpC* | This work |
| PTargetF-P3 | *pMB1 aadA* sgRNA- butyryl-CoA: acetate: CoA transferase | This work |

Table S2 Primers and N20 sequence under in this study

| Primers and N_2_ | Sequence ( 5＇-3＇) |
| --- | --- |
| **Primers used for the construction of pTargetF vectors** | |
| *skfA*-for | CATGCCATGGATAACATAATGGACCGTCTTTTTGACG |
| *skfA*-rev | GGGAAGCTTCGTTTTATGAAGATTTTTGTTTTTTGATAACAG |
| *skfA*-1-for | ATGAAAAGAAACCAAAAAGAATGGG |
| *skfA*-1-rev | TTAAATAGCTCTCATAGCAGGATGCG |
| *sdpC*-for | TTGAAAAGTAAATTACTTAGGCTATTGATTGTTTCC |
| *sdpC*-rev | TTATTGATGAATCAATTTTAGGGTTTTTGCAGC |
| *sdpC*-1-for | GATAATATTCAGCATTGGTATTGTAAGTTTTGGC |
| *sdpC*-1-rev | CCTCTAAAGTACTTGTAGTGTGTGGTTTTATAATAGCTG |
| B-CoA: A CoA-for | GGATCCATTTTACATTTTTAGAAATGGGC |
| B-CoA: A CoA-rev | AAGCTTGTGTCCGCAATCGCC |
| **N_20_ sequence for gRNA** | |
| *skfA* edition | TGGGAATCTGTGAGTAAAAA |
| BCoAT edition | TTCATTAGTAGGACTCTCTA |
